# Effects of maternal poor ovarian response on the reproductive endocrine profiles of the next generation: a prospective cohort study in China

**DOI:** 10.1093/hropen/hoaf019

**Published:** 2025-03-28

**Authors:** Wanbing Feng, Yujia Ren, Jiayi Zhou, Hanbing Zhu, Han Zhao, Yingying Qin, Jing Li, Mingdi Xia, Lihong Xu, Mei Li, Huidan Wang, Linlin Cui, Zi-Jiang Chen

**Affiliations:** State Key Laboratory of Reproductive Medicine and Offspring Health, Center for Reproductive Medicine, Institute of Women, Children and Reproductive Health, Shandong University, Jinan, Shandong, China; National Research Center for Assisted Reproductive Technology and Reproductive Genetics, Shandong University, Jinan, Shandong, China; Key Laboratory of Reproductive Endocrinology (Shandong University), Ministry of Education, Jinan, Shandong, China; Shandong Technology Innovation Center for Reproductive Health, Jinan, Shandong, China; Shandong Provincial Clinical Research Center for Reproductive Health, Jinan, Shandong, China; Shandong Key Laboratory of Reproductive Research and Birth Defect Prevention, Jinan, Shandong, China; Research Unit of Gametogenesis and Health of ART-Offspring, Chinese Academy of Medical Sciences (No. 2021RU001), Jinan, Shandong, China; State Key Laboratory of Reproductive Medicine and Offspring Health, Center for Reproductive Medicine, Institute of Women, Children and Reproductive Health, Shandong University, Jinan, Shandong, China; National Research Center for Assisted Reproductive Technology and Reproductive Genetics, Shandong University, Jinan, Shandong, China; Key Laboratory of Reproductive Endocrinology (Shandong University), Ministry of Education, Jinan, Shandong, China; Shandong Technology Innovation Center for Reproductive Health, Jinan, Shandong, China; Shandong Provincial Clinical Research Center for Reproductive Health, Jinan, Shandong, China; Shandong Key Laboratory of Reproductive Research and Birth Defect Prevention, Jinan, Shandong, China; Research Unit of Gametogenesis and Health of ART-Offspring, Chinese Academy of Medical Sciences (No. 2021RU001), Jinan, Shandong, China; State Key Laboratory of Reproductive Medicine and Offspring Health, Center for Reproductive Medicine, Institute of Women, Children and Reproductive Health, Shandong University, Jinan, Shandong, China; National Research Center for Assisted Reproductive Technology and Reproductive Genetics, Shandong University, Jinan, Shandong, China; Key Laboratory of Reproductive Endocrinology (Shandong University), Ministry of Education, Jinan, Shandong, China; Shandong Technology Innovation Center for Reproductive Health, Jinan, Shandong, China; Shandong Provincial Clinical Research Center for Reproductive Health, Jinan, Shandong, China; Shandong Key Laboratory of Reproductive Research and Birth Defect Prevention, Jinan, Shandong, China; Research Unit of Gametogenesis and Health of ART-Offspring, Chinese Academy of Medical Sciences (No. 2021RU001), Jinan, Shandong, China; State Key Laboratory of Reproductive Medicine and Offspring Health, Center for Reproductive Medicine, Institute of Women, Children and Reproductive Health, Shandong University, Jinan, Shandong, China; National Research Center for Assisted Reproductive Technology and Reproductive Genetics, Shandong University, Jinan, Shandong, China; Key Laboratory of Reproductive Endocrinology (Shandong University), Ministry of Education, Jinan, Shandong, China; Shandong Technology Innovation Center for Reproductive Health, Jinan, Shandong, China; Shandong Provincial Clinical Research Center for Reproductive Health, Jinan, Shandong, China; Shandong Key Laboratory of Reproductive Research and Birth Defect Prevention, Jinan, Shandong, China; Research Unit of Gametogenesis and Health of ART-Offspring, Chinese Academy of Medical Sciences (No. 2021RU001), Jinan, Shandong, China; State Key Laboratory of Reproductive Medicine and Offspring Health, Center for Reproductive Medicine, Institute of Women, Children and Reproductive Health, Shandong University, Jinan, Shandong, China; National Research Center for Assisted Reproductive Technology and Reproductive Genetics, Shandong University, Jinan, Shandong, China; Key Laboratory of Reproductive Endocrinology (Shandong University), Ministry of Education, Jinan, Shandong, China; Shandong Technology Innovation Center for Reproductive Health, Jinan, Shandong, China; Shandong Provincial Clinical Research Center for Reproductive Health, Jinan, Shandong, China; Shandong Key Laboratory of Reproductive Research and Birth Defect Prevention, Jinan, Shandong, China; Research Unit of Gametogenesis and Health of ART-Offspring, Chinese Academy of Medical Sciences (No. 2021RU001), Jinan, Shandong, China; State Key Laboratory of Reproductive Medicine and Offspring Health, Center for Reproductive Medicine, Institute of Women, Children and Reproductive Health, Shandong University, Jinan, Shandong, China; National Research Center for Assisted Reproductive Technology and Reproductive Genetics, Shandong University, Jinan, Shandong, China; Key Laboratory of Reproductive Endocrinology (Shandong University), Ministry of Education, Jinan, Shandong, China; Shandong Technology Innovation Center for Reproductive Health, Jinan, Shandong, China; Shandong Provincial Clinical Research Center for Reproductive Health, Jinan, Shandong, China; Shandong Key Laboratory of Reproductive Research and Birth Defect Prevention, Jinan, Shandong, China; Research Unit of Gametogenesis and Health of ART-Offspring, Chinese Academy of Medical Sciences (No. 2021RU001), Jinan, Shandong, China; State Key Laboratory of Reproductive Medicine and Offspring Health, Center for Reproductive Medicine, Institute of Women, Children and Reproductive Health, Shandong University, Jinan, Shandong, China; National Research Center for Assisted Reproductive Technology and Reproductive Genetics, Shandong University, Jinan, Shandong, China; Key Laboratory of Reproductive Endocrinology (Shandong University), Ministry of Education, Jinan, Shandong, China; Shandong Technology Innovation Center for Reproductive Health, Jinan, Shandong, China; Shandong Provincial Clinical Research Center for Reproductive Health, Jinan, Shandong, China; Shandong Key Laboratory of Reproductive Research and Birth Defect Prevention, Jinan, Shandong, China; Research Unit of Gametogenesis and Health of ART-Offspring, Chinese Academy of Medical Sciences (No. 2021RU001), Jinan, Shandong, China; State Key Laboratory of Reproductive Medicine and Offspring Health, Center for Reproductive Medicine, Institute of Women, Children and Reproductive Health, Shandong University, Jinan, Shandong, China; National Research Center for Assisted Reproductive Technology and Reproductive Genetics, Shandong University, Jinan, Shandong, China; Key Laboratory of Reproductive Endocrinology (Shandong University), Ministry of Education, Jinan, Shandong, China; Shandong Technology Innovation Center for Reproductive Health, Jinan, Shandong, China; Shandong Provincial Clinical Research Center for Reproductive Health, Jinan, Shandong, China; Shandong Key Laboratory of Reproductive Research and Birth Defect Prevention, Jinan, Shandong, China; Research Unit of Gametogenesis and Health of ART-Offspring, Chinese Academy of Medical Sciences (No. 2021RU001), Jinan, Shandong, China; Department of Gynaecology and Obstetrics, Shandong Provincial Hospital Affiliated to Shandong First Medical University, Jinan, Shandong, China; National Research Center for Assisted Reproductive Technology and Reproductive Genetics, Shandong University, Jinan, Shandong, China; Key Laboratory of Reproductive Endocrinology (Shandong University), Ministry of Education, Jinan, Shandong, China; Shandong Technology Innovation Center for Reproductive Health, Jinan, Shandong, China; Shandong Provincial Clinical Research Center for Reproductive Health, Jinan, Shandong, China; Shandong Key Laboratory of Reproductive Research and Birth Defect Prevention, Jinan, Shandong, China; Research Unit of Gametogenesis and Health of ART-Offspring, Chinese Academy of Medical Sciences (No. 2021RU001), Jinan, Shandong, China; The Second Hospital, State Key Laboratory of Reproductive Medicine and Offspring Health, Center for Reproductive Medicine, Institute of Women, Children and Reproductive Health, Shandong University, Jinan, Shandong, China; State Key Laboratory of Reproductive Medicine and Offspring Health, Center for Reproductive Medicine, Institute of Women, Children and Reproductive Health, Shandong University, Jinan, Shandong, China; National Research Center for Assisted Reproductive Technology and Reproductive Genetics, Shandong University, Jinan, Shandong, China; Key Laboratory of Reproductive Endocrinology (Shandong University), Ministry of Education, Jinan, Shandong, China; Shandong Technology Innovation Center for Reproductive Health, Jinan, Shandong, China; Shandong Provincial Clinical Research Center for Reproductive Health, Jinan, Shandong, China; Shandong Key Laboratory of Reproductive Research and Birth Defect Prevention, Jinan, Shandong, China; Research Unit of Gametogenesis and Health of ART-Offspring, Chinese Academy of Medical Sciences (No. 2021RU001), Jinan, Shandong, China; National Research Center for Assisted Reproductive Technology and Reproductive Genetics, Shandong University, Jinan, Shandong, China; Key Laboratory of Reproductive Endocrinology (Shandong University), Ministry of Education, Jinan, Shandong, China; Shandong Technology Innovation Center for Reproductive Health, Jinan, Shandong, China; Shandong Provincial Clinical Research Center for Reproductive Health, Jinan, Shandong, China; Shandong Key Laboratory of Reproductive Research and Birth Defect Prevention, Jinan, Shandong, China; Research Unit of Gametogenesis and Health of ART-Offspring, Chinese Academy of Medical Sciences (No. 2021RU001), Jinan, Shandong, China; The Second Hospital, State Key Laboratory of Reproductive Medicine and Offspring Health, Center for Reproductive Medicine, Institute of Women, Children and Reproductive Health, Shandong University, Jinan, Shandong, China; State Key Laboratory of Reproductive Medicine and Offspring Health, Center for Reproductive Medicine, Institute of Women, Children and Reproductive Health, Shandong University, Jinan, Shandong, China; National Research Center for Assisted Reproductive Technology and Reproductive Genetics, Shandong University, Jinan, Shandong, China; Key Laboratory of Reproductive Endocrinology (Shandong University), Ministry of Education, Jinan, Shandong, China; Shandong Technology Innovation Center for Reproductive Health, Jinan, Shandong, China; Shandong Provincial Clinical Research Center for Reproductive Health, Jinan, Shandong, China; Shandong Key Laboratory of Reproductive Research and Birth Defect Prevention, Jinan, Shandong, China; Research Unit of Gametogenesis and Health of ART-Offspring, Chinese Academy of Medical Sciences (No. 2021RU001), Jinan, Shandong, China; Shanghai Key Laboratory for Assisted Reproduction and Reproductive Genetics, Shanghai, China; Department of Reproductive Medicine, Ren Ji Hospital, Shanghai Jiao Tong University School of Medicine, Shanghai, China

**Keywords:** poor ovarian response, offspring health, ovarian reserve, anti-Müllerian hormone, reproductive endocrine, POSEIDON criteria

## Abstract

**STUDY QUESTION:**

Do offspring born to mothers with poor ovarian response (POR) have alterations in their reproductive endocrine profile at 2–6 years of age compared to those born to mothers with normal ovarian response?

**SUMMARY ANSWER:**

Female offspring born to young mothers (<35 years) with expected POR were more likely to have low serum anti-Müllerian hormone (AMH) levels in childhood.

**WHAT IS KNOWN ALREADY:**

POR affects 32–43% of women in infertility clinics. Genetic susceptibility and potentially adverse intrauterine environments pose threats to the next generation. However, there is currently no direct evidence of intergenerational reproductive effects associated with POR.

**STUDY DESIGN, SIZE, DURATION:**

We conducted a prospective cohort study to investigate the intergenerational effects of maternal POR on reproductive endocrine health of offspring. Data were obtained from ‘Assisted Reproductive Technology-born KIDs (ARTKID)’, a birth cohort established in 2013 at a tertiary care center in China. A total of 3103 offspring, aged 2–6, born between 2013 and 2019, were recruited and included in our study until 2021. The exposed offspring conceived by ART were classified into four groups based on their mothers’ categorization using the Patient-Oriented Strategies Encompassing IndividualizeD Oocyte Number (POSEIDON) criteria. The unexposed offspring were born to mothers with normal ovarian response after ART.

**PARTICIPANTS/MATERIALS, SETTING, METHODS:**

Offspring conceived by ART provided blood samples at 2–6 years for the assessment of reproductive endocrine parameters. Mean difference and 95% CI were obtained based on a linear mixed model. The adjusted model accounted for paternal age, maternal age, offspring age, paternal smoking, use of ICSI, and frozen embryo transfer.

**MAIN RESULTS AND THE ROLE OF CHANCE:**

Female offspring born to young mothers with expected POR (POSEIDON Group 3) had lower AMH and PRL (prolactin) levels in childhood compared to controls (AMH: adjusted mean difference [AMD] = −0.64, 95% CI = −1.10, −0.18; PRL: AMD = −1.59, 95% CI = −2.97, −0.21). Female offspring born to older mothers (≥35 years) with expected POR (POSEIDON Group 4) showed a decreasing trend in AMH levels, though this difference was not statistically significant compared to controls [AMD = −0.60, 95% CI = −1.31, −0.12]. Female offspring born to young mothers with unexpected POR (POSEIDON Group 1) had lower DHEA-S (dehydroepiandrosterone sulfate) levels than controls [AMD = −1.38, 95% CI = −2.58, −0.17]. In contrast, male offspring born to POR mothers showed similar reproductive endocrine profiles as controls.

**LIMITATIONS, REASONS FOR CAUTION:**

The offspring were aged 2–6 years, limiting the ability to assess comprehensive reproductive phenotypic changes. Longer follow-up studies are necessary.

**WIDER IMPLICATIONS OF THE FINDINGS:**

The potential effects of maternal POR on reproductive endocrine profiles of offspring may be primarily linked to ovarian reserve. Genetic susceptibility, hypoandrogenism, and other intrauterine environmental factors may be probable explanations for reduction in AMH levels observed in female offspring born to young mothers with expected POR.

**STUDY FUNDING/COMPETING INTEREST(S):**

This study was supported by the National Key Research and Development Program of China (2022YFC2703000, 2022YFC2704404, 2024YFC2706902, 2022YFC2702905, 2024YFC2706700), CAMS Innovation Fund for Medical Sciences (2021-I2M-5-001), Shandong Provincial Natural Science Foundation (ZR2022JQ33), the Fundamental Research Funds of Shandong University (2023QNTD004), the National Special Support Program for High-level Talents, the Health Science and Technology Innovation Team Construction Project of Shandong Province, and the Taishan Scholars Program for Young Experts of Shandong Province (tsqn201909195). The authors declare that they have no competing interests.

**TRIAL REGISTRATION NUMBER:**

N/A.

WHAT DOES THIS MEAN FOR PATIENTS? Poor ovarian response (POR) is a condition that affects nearly half of women undergoing fertility treatment and is one of the main obstacles to having successful pregnancies. POR means that a woman’s ovaries don’t respond well to fertility treatments, producing fewer eggs. Studies have suggested that both genetic factors and environmental influences could affect the reproductive health of future generations, regardless of whether they are sons or daughters. This raises concerns about the potential effect of a mother’s POR condition on the fertility of her children.

This study, conducted in China, focused on mother–child pairs. Over several years, it was found that daughters born to young mothers (age <35 years) with low ovarian reserve (a condition where fewer eggs remain in the ovaries, a type of POR) were likely to have lower levels of a hormone called anti-Müllerian hormone (which is used to measure a woman’s fertility) than daughters of women not suffering from POR. This suggests that a mother’s fertility could affect her daughter’s future fertility, possibly leading to challenges in having children later in life. No significant effects were found in male children.

For these patients (age <35 years with low ovarian reserve), these findings suggest that fertility problems might affect future generations, particularly daughters. Further studies are needed to track how their daughters’ fertility may change as they grow older.

## Introduction

Poor ovarian response (POR) is one of the most challenging obstacles in infertility clinics. The condition is characterized by poor response to ovarian stimulation, affecting 32–43% of women undergoing IVF according to the POSEIDON (Patient-Oriented Strategies Encompassing IndividualizeD Oocyte Number) criteria (Li *et al.*, 2019; Esteves *et al.*, 2021a; Cedars, 2022). The POSEIDON criteria classify patients with POR into four groups based on their age, ovarian reserve markers such as anti-Müllerian hormone (AMH) and/or antral follicle count (AFC), and the number of oocytes retrieved if the patient has previously undergone standard ovarian stimulation, with each group representing different pathophysiological conditions (Alviggi *et al.*, 2016). The POSEIDON criteria stratify women with POR into two main categories: ‘unexpected POR’ (groups 1 and 2 with normal ovarian reserve) and ‘expected POR’ (groups 3 and 4 with decreased ovarian reserve). Due to the limited number of oocytes retrieved, the cumulative live birth rate (CLBR) of women with POR remains substantially low (Yang *et al.*, 2020; Esteves *et al.*, 2021b; Yan *et al.*, 2023). However, in addition to considering successful delivery outcomes, it is essential to also focus on the health of their offspring.

Accumulating evidence has suggested that, owing to decreased oocyte quantity and quality, women with POR show adverse pregnancy outcomes compared to the normal responders, including higher aneuploid blastocyst and clinical miscarriage rates (Sunkara *et al.*, 2014; Shahine *et al.*, 2016; Jaswa *et al.*, 2021; Bahadur *et al.*, 2023). Observational studies have found that women with diminished ovarian reserve (AFC ≤ 6/FSH ≥ 10 IU/L and AMH < 1.2 ng/ml) have a higher incidence of pregnancy with obstetric complications, including hypertensive disorders of pregnancy, multiple placental fetal vascular lesions, and preterm birth (Woldringh *et al.*, 2006; Yang *et al.*, 2019; Han *et al.*, 2021; Ganer Herman *et al.*, 2023). Moreover, adverse early-life factors have been associated with the reproductive health of both female and male offspring (Swamy *et al.*, 2008; Dior *et al.*, 2021; van der Pal *et al.*, 2021; Laru *et al.*, 2022). However, the long-term reproductive health of offspring born to mothers with POR, as defined by the POSEIDON criteria, has received limited attention.

It has been recognized that there are intergenerational trends in the causes and outcomes of infertility (Woolner and Bhattacharya, 2023). Several single-gene mutations, genetic polymorphisms, and epigenetic changes are associated with POR, supporting the genetic susceptibility of this condition (Greene *et al.*, 2014; Nesbit *et al.*, 2020; Olsen *et al.*, 2021). Women in expected POR groups with low ovarian reserve are more likely to experience earlier menopause (Freeman *et al.*, 2012). Previous population studies have suggested that mothers who experienced menopause earlier may have daughters with lower ovarian reserves (Rosen *et al.*, 2010; Bentzen *et al.*, 2013). Moreover, women with POR often exhibit a deficiency in basal testosterone (T) (Qin *et al.*, 2011; Xiao *et al.*, 2016), which may persist into pregnancy and result in adverse intrauterine environmental changes. Additionally, the use of antiandrogens during late gestation has been shown to adversely impact reproductive organ development in male rat offspring (McIntyre *et al.*, 2001; Pallarés *et al.*, 2014; Mentessidou *et al.*, 2021). All these findings imply possible unfavorable reproductive health changes in offspring born to mothers with POR. However, to the best of our knowledge, no direct evidence of the intergenerational effects of POR currently exists.

Therefore, we conducted a prospective cohort study to explore the impact of maternal POR on the reproductive endocrine health of their offspring from the ages of 2–6 years. The study aimed to provide valuable insights into the guidance for monitoring and managing long-term reproductive health in individuals born to mothers with POR.

## Materials and methods

### Ethical approval

The study was approved by the Reproductive Medicine Ethics Committee, Hospital for Reproductive Medicine Affiliated to Shandong University. All parents signed informed consent forms with the assent of the child.

### Study design and participants

Participating offspring are part of the ‘Assisted Reproductive Technology-born KIDs (ARTKID)’ cohort, a longitudinal prospective birth cohort of offspring born via ART at the Institute of Women, Children and Reproductive Health, Shandong University, China. The details of the cohort design have been presented in our previous article (Zhou *et al.*, 2024). The ‘ARTKID’ cohort focuses on the growth, endocrine, and metabolic health of offspring. Offspring who did not undergo growth assessments were classified as lost to follow-up. During face-to-face visits, children’s height and weight were measured, relevant questionnaires were completed, and blood samples were collected.

The offspring included in this study were born between 2013 and 2019, and the follow-up period lasted until August 2021. The study included singleton offspring conceived via IVF or ICSI. Mothers who underwent preimplantation genetic testing, gamete intrauterine transfer, *in vitro* maturation (IVM), sperm donation, sperm freezing, oocyte donation, or minimal or natural ovarian stimulation cycles were excluded from the analysis. Also excluded were those with parental chromosomal abnormalities, maternal polycystic ovarian syndrome (Li *et al.*, 2020), or missing AMH values.

**Figure 1.**
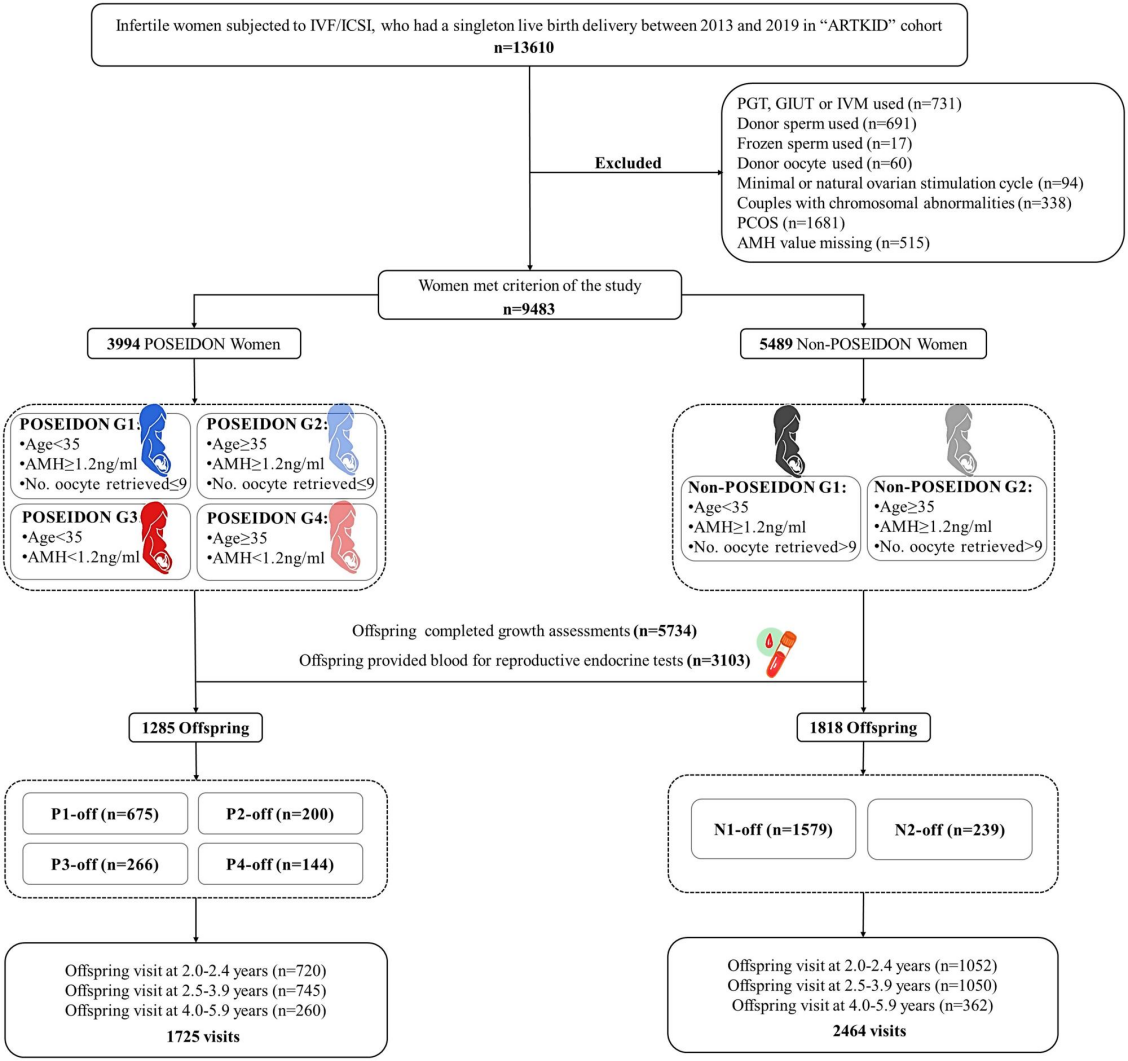
**Flowchart of the study.** Blue represents pregnant mothers with unexpected POR. Red represents pregnant mothers with expected POR. Gray represents pregnant mothers without POR. Pale shades indicate the older individuals within the same group. AMH, anti-Müllerian hormone; ARTKID, Assisted Reproductive Technology-born KIDs; GIUT, gamete intrauterine transfer; PGT, preimplantation genetic testing; POR, poor ovarian response; POSEIDON, Patient-Oriented Strategies Encompassing IndividualizeD Oocyte Number; POSEIDON G1, POSEIDON Group 1; POSEIDON G2, POSEIDON Group 2; POSEIDON G3, POSEIDON Group 3; POSEIDON G4, POSEIDON Group 4; P1-off, offspring born to mothers in POSEIDON Group 1; P2-off, offspring born to mothers in POSEIDON Group 2; P3-off, offspring born to mothers in POSEIDON Group 3; P4-off, offspring born to mothers in POSEIDON Group 4; N1-off, offspring born to mothers in Non-POSEIDON Group 1; N2-off, offspring born to mothers in Non-POSEIDON Group 2.

In total, 9483 mother–offspring pairs from the entire ‘ARTKID’ cohort met the inclusion criteria. Among these, 5734 individuals completed growth assessments, resulting in a follow-up rate of 60.5% (5734/9483) as of August 2021. Furthermore, 1285 children born to mothers with POR and 1818 children born to mothers without POR provided blood samples for the assessment of reproductive endocrine parameters and were included in the present analysis. Our longitudinal study utilized a follow-up design conducted at three specific age ranges: 2.0–2.4 years, 2.5–3.9 years, and 4.0–5.9 years. Each offspring was required to be followed up at least once. Most offspring participated only once, while some returned for follow-up assessments twice (836 children) or three times (125 children). The mean duration of follow-up across the cohort was 2.9 years, with a SD of 0.9 years and a range from 2 to 6 years. The flowchart illustrating this process is presented in Fig. 1.

### Exposure

The main exposure measure was maternal POR. POR had been diagnosed and classified into four groups based on the POSEIDON criteria (Alviggi *et al.*, 2016)—(i) POSEIDON Group 1: age <35 years, AMH ≥1.2 ng/ml, and a standard ovarian stimulation with ≤9 oocytes retrieved; (ii) POSEIDON Group 2: age ≥35 years, AMH ≥1.2 ng/ml, and a standard ovarian stimulation with ≤9 oocytes retrieved; (iii) POSEIDON Group 3: age <35 years and AMH <1.2 ng/ml; (iv) POSEIDON Group 4: age ≥35 years and AMH <1.2 ng/ml. The comparison groups included offspring whose mothers had an adequate ovarian reserve and a normal response to a standard ovarian stimulation: (i) Non-POSEIDON Group 1: age <35 years, AMH ≥1.2 ng/ml, and >9 oocytes retrieved; (ii) Non-POSEIDON Group 2: age ≥35 years, AMH ≥1.2 ng/ml, and >9 oocytes retrieved. We used serum AMH as the ovarian reserve biomarker because recent studies suggested that it may be more accurate and robust than AFC (Fleming *et al.*, 2015; Iliodromiti *et al.*, 2015; Nelson *et al.*, 2015).

The exposed groups consisted of offspring conceived through ART and born to POR mothers, including those born to POSEIDON Group 1 mothers (P1-off), POSEIDON Group 2 mothers (P2-off), POSEIDON Group 3 mothers (P3-off), and POSEIDON Group 4 mothers (P4-off). The control groups consisted of offspring conceived through ART and born to mothers with normal ovarian response, including those born to Non-POSEIDON Group 1 (N1-off) and Non-POSEIDON Group 2 (N2-off). Taking into account the impact of maternal age on offspring health, the N1-off group was the control group for P1-off and P3-off groups (groups of mothers <35 years), while the N2-off group was the control group for P2-off and P4-off groups (groups of mothers ≥35 years).

Maternal basic endocrinological profiles and AFC were tested during the first 3 days of the menstrual cycle within 1 year prior to the IVF/ICSI cycle. The AFC was defined as the count of follicles with a diameter of 2–9 mm in both ovaries. Maternal estradiol (E_2_) levels were tested on hCG trigger day, including both fresh cycles and the oocyte retrieval cycles that led to embryos used in frozen embryo transfer (FET) cycles. In both fresh and frozen embryo cycles, the stimulation protocols included GnRH agonist long protocols, GnRH agonist short protocols, and GnRH antagonist protocols. Endometrial preparation in frozen cycles included natural and artificial cycles. Blood samples were collected at the end of the first trimester (11–13 gestational weeks) for E_2_, DHEA-S (dehydroepiandrosterone sulfate), and T tests in mothers. All blood samples were stored at −80°C until detection.

### Outcomes

Reproductive endocrine profiles were assessed in offspring aged 2–6 years, with fasting blood samples collected at these age stages: 2.0–2.4 years, 2.5–3.9 years, and 4.0–5.9 years. All samples were collected by well-trained pediatricians and nurses and stored at −80°C until analysis. The hormones included FSH, LH, E_2_, T, prolactin (PRL), DHEA-S, and AMH. AMH was tested using an ELISA (Ansh Labs, Webster, TX, USA). FSH, LH, E_2_, T, PRL, and DHEA-S were tested by chemiluminescence immunoassays (Roche Diagnostics, Mannheim, Germany) with intra- and inter-assay coefficients of variation of less than 10%. All hormones were tested by laboratory physicians, with regular quality control measures implemented to ensure data accuracy.

### Covariates

Potential confounders were defined based on prior literature reporting of indicators associated with offspring’s long-term reproductive health outcomes, including maternal age (Basso *et al.*, 2018), paternal age (Sartorius and Nieschlag, 2010; Ashapkin *et al.*, 2023), offspring age (Cui *et al.*, 2016), paternal smoking (Fraser *et al.*, 2013), use of ICSI (Belva *et al.*, 2016; Belva *et al.*, 2017; Catford *et al.*, 2020), and FET (Shaw *et al.*, 2012; Jiang *et al.*, 2017). Parental characteristics were obtained through standardized measurements and questionnaires administered during face-to-face interviews prior to the IVF/ICSI cycle. In both fresh and frozen embryo cycles, parental ages were calculated based on the date of parental birth and the date of oocyte retrieval. ART characteristics were recorded in the medical records during the IVF/ICSI cycle.

### Statistics

The statistical analyses were conducted using the R statistical software, version 4.2.3 (R Foundation for Statistical Computing, Vienna, Austria). Continuous characteristic variables were assessed for normality. For normally distributed variables, Student’s *t*-test was employed for analysis, while the Mann–Whitney *U* test was used for those that did not follow a normal distribution. Categorical characteristic variables were compared using either the chi-square test or Fisher’s exact test. The association between maternal POR and reproductive endocrine profiles in offspring was assessed using a linear mixed-effects regression model. Reproductive endocrine profiles were evaluated in offspring at three specific age ranges: 2.0–2.4 years, 2.5–3.9 years, and 4.0–5.9 years. Each offspring had 1–3 repeated measurements. If more than one sample was taken from a child, all blood samples were collected, and all available reproductive endocrine hormone measurements were included in the analysis. We did not exclude any sample based on the number of follow-ups, ensuring that all available data were utilized for a comprehensive analysis. The mixed-effects model allowed us to account for the potential confounding effects of repeated measurements taken from the same child at different time points. A *P*-value of <0.05 was regarded as statistically significant.

## Results

### Baseline characteristics of offspring from mothers with unexpected POR

The parental ages of offspring born to mothers with unexpected POR (POSEIDON Groups 1 and 2) were older than those of their non-POR counterparts. Maternal BMI was higher in POSEIDON Group 1 than in controls, with no significant differences in Group 2. Paternal BMI, smoking status, and parental education levels were comparable between groups. Unexpected POR mothers showed lower AFC, AMH, and T and higher FSH compared to controls. Additionally, maternal LH was higher and E_2_ was lower in POSEIDON Group 1 than in controls. These characteristics align with unexpected POR traits (Table 1).

**Table 1.** Parental and ART characteristics of study participants.

	POSEIDON-off Groups				Non-POSEIDON-off Groups	
	P1-off n = 675	P2-off n = 200	P3-off n = 266	P4-off n = 144	N1-off n = 1579	N2-off n = 239
**Parental characteristics**						
Paternal age, years	30.5 ± 3.9*	38.2 ± 3.7	31.2 ± 3.9*	38.4 ± 4.6	29.9 ± 3.8	37.9 ± 4.2
Maternal age, years	29.6 ± 3.1*	38.1 ± 2.2^#^	30.3 ± 3.1*	38.2 ± 2.2^#^	29.1 ± 3	37.4 ± 2
Paternal BMI, kg/m^2^	25.7 ± 3.9	26.6 ± 3.3	26.3 ± 4.1	26.8 ± 3.3	25.8 ± 4.2	27 ± 3.8
Maternal BMI, kg/m^2^	23.3 ± 3.7*	24.3 ± 4	23.2 ± 3.4	24.3 ± 3.6	22.9 ± 3.5	24.3 ± 3.8
Paternal education level (college or higher), n (%)	273 (40.4)	70 (35)	108 (40.6)	60 (41.7)	638 (40.4)	97 (40.6)
Maternal education level (college or higher), n (%)	252 (37.3)	61 (30.5)	97 (36.5)	46 (31.9)	588 (37.2)	83 (34.7)
Paternal smoking, n (%)	229 (33.9)	57 (28.5)	92 (34.6)	44 (30.6)	521 (33)	53 (22.2)
Maternal AFC	11.8 ± 5.4*	9.9 ± 4.1^#^	9 ± 4.1*	7 ± 3^#^	15.1 ± 5.8	13.6 ± 5
Maternal basal endocrine						
AMH, ng/ml	3.8 ± 2.7*	3 ± 2^#^	0.7 ± 0.3*	0.7 ± 0.3^#^	5.6 ± 3.6	4.3 ± 2.5
FSH, IU/L	7 ± 1.7*	7.4 ± 1.9^#^	7.9 ± 3.5*	9.2 ± 3.7^#^	6.2 ± 1.4	6.5 ± 1.6
LH, IU/L	5 ± 2.8*	4.7 ± 2.3	4.5 ± 2.4*	4.7 ± 2.1	5.4 ± 2.8	4.9 ± 3.4
E_2_, pg/ml	33.7 ± 12.8*	36.1 ± 13.6	33.4 ± 14*	35.8 ± 15.1	37.4 ± 21.5	36.9 ± 27.6
T, ng/dl	24.3 ± 11.4*	20.4 ± 9.8^#^	20.8 ± 10.2*	18.8 ± 10.3^#^	25.8 ± 13.3	22.6 ± 10.8
PRL, ng/L	18.6 ± 19.9	16.1 ± 8.6	15.7 ± 7.3*	14.5 ± 6.3	18 ± 9.5	15.6 ± 7.8
DHEA-S, ug/dl	251.3 ± 92.7	206.6 ± 73.2	240.8 ± 95.2	211.8 ± 80.7	252.9 ± 92.7	214.7 ± 85.5
**ART characteristics**						
Maternal total gonadotropin dose, IU	1969.6 ± 924.8*	2078.4 ± 832.2	2169.2 ± 1102.2*	2571.6 ± 1183.7^#^	1744.9 ± 759.9	2066.2 ± 861.6
Maternal E_2_ levels on hCG trigger day, pg/ml	2463.2 ± 1232.9*	2470.9 ± 1163.1^#^	2695.3 ± 1666.2*	2137.8 ± 1420.6^#^	4533.1 ± 2052.7	4365.1 ± 1863.6
No. of oocytes retrieved	6.8 ± 1.9*	6.2 ± 2^#^	8.4 ± 4.8*	5.8 ± 3.6^#^	15.6 ± 4.7	14.3 ± 3.8
ICSI used, n (%)	191 (28.3)*	52 (26)	72 (27.1)*	34 (23.6)	543 (34.4)	61 (25.5)
Frozen embryo transfer, n (%)	244 (36.1)*	70 (35)^**#**^	92 (34.6)*	47 (32.6)^**#**^	1013 (64.2)	137 (57.3)

Data are presented as mean ± SD or n (%).

The reference group for the P1-off and P3-off groups was the N1-off group (*P <* *0.05).

The reference group for the P2-off and P4-off groups was the N2-off group (^#^*P *<* *0.05).

Student’s *t*-test was used for analysis of normally distributed variables, while the Mann–Whitney *U* test was applied to variables that did not follow a normal distribution.

AFC, antral follicle count; AMH, anti-Müllerian hormone; ART, DHEA-S, dehydroepiandrosterone sulfate; E_2,_ estradiol; N1-off, offspring born to Non-POSEIDON Group 1 mothers; N2-off, offspring born to Non-POSEIDON Group 2 mothers; POSEIDON, Patient-Oriented Strategies Encompassing IndividualizeD Oocyte Number; P1-off, offspring born to POSEIDON Group 1 mothers; P2-off, offspring born to POSEIDON Group 2 mothers; P3-off, offspring born to POSEIDON Group 3 mothers; P4-off, offspring born to POSEIDON Group 4 mothers; PRL, prolactin; T, testosterone.

**Figure 2.**
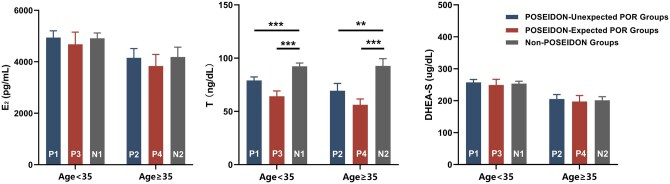
**Serum endocrinological status of pregnant mothers at the end of first trimester.** All data are presented as mean ± SEM. POSEIDON-Unexpected POR groups include P1 and P2, while POSEIDON-Expected POR groups include P3 and P4. The Non-POSEIDON groups refer to infertile women with a normal ovarian response, including N1 and N2 categories. Considering age of mothers, the reference group for the P1 and P3 groups was the N1 group (age <35 years), whereas the reference group for the P2 and P4 groups was the N2 group (age ≥35 years). The Mann–Whitney *U* test was used to analyze the significance of the data. **P < 0.01, ***P < 0.001. P1, n = 188; P2, n = 62; P3, n = 58; P4, n = 47; N1, n = 276; and N2, n = 62. DHEA-S, dehydroepiandrosterone sulfate; E_2_, estradiol; POR, poor ovarian response; POSEIDON, Patient-Oriented Strategies Encompassing IndividualizeD Oocyte Number; P1, POSEIDON Group 1; P2, POSEIDON Group 2; P3, POSEIDON Group 3; P4, POSEIDON Group 4; N1, Non-POSEIDON Group 1; N2, Non-POSEIDON Group 2; T, testosterone.

During the IVF/ICSI cycle, POSEIDON Group 1 mothers required higher gonadotropin doses. Unexpected POR mothers had lower E_2_ levels on hCG trigger day, and approximately half the number of oocytes were retrieved compared to their non-POR counterparts. Additionally, POSEIDON Group 1 mothers had fewer ICSI cycles. Unexpected POR mothers underwent fewer FET cycles (Table 1). During early pregnancy, T levels were lower in unexpected POR mothers, with no significant changes in E_2_ and DHEA-S levels (Fig. 2).

### Baseline characteristics of offspring from mothers with expected POR

Offspring born to mothers with expected POR (POSEIDON Groups 3 and 4) also had older parental ages than controls. Parental BMI, education levels, and paternal smoking status were comparable. Ovarian reserve was more diminished in expected POR mothers, who had significantly lower AFC, AMH, and T and higher FSH levels. Maternal LH was higher, while E_2_ and PRL levels were lower in POSEIDON Group 3 compared to controls.

During their IVF/ICSI cycles, expected POR mothers used more gonadotropin and showed lower E_2_ levels on the hCG trigger day than controls. The number of oocytes retrieved was more than 2-fold lower compared to non-POR mothers. POSEIDON Group 3 mothers underwent fewer ICSI cycles, and expected POR mothers had fewer FET cycles (Table 1). Early pregnancy T levels were also lower in expected POR mothers (Fig. 2).

### Comparison of baseline characteristics between included and excluded offspring in the ‘ARTKID’ cohort

We compared the baseline characteristics of offspring included in the analysis with those excluded from the whole ‘ARTKID’ cohort (Supplementary Table S1). Importantly, our sample size is relatively large. Although we observed differences in parental age, paternal smoking status, maternal E_2_ levels on the hCG trigger day, and the number of oocytes retrieved, the actual differences were relatively minor. For example, the difference in maternal age between the included and excluded offspring was only 0.44 years.

### The effects of maternal unexpected POR on reproductive endocrine characteristics of offspring

Compared to the control group, female offspring born to mothers in POSEIDON Group 2 showed higher FSH levels (mean difference [MD] = 0.43, 95% CI = 0.04, 0.82, *P* = 0.031). However, the differences were no longer significant after adjusting parental age, offspring age, paternal smoking, ICSI, and FET use (adjusted mean difference [AMD] = 0.37, 95% CI = −0.01, 0.74, *P* = 0.054) (Tables 2 and 3 and Supplementary Table S2).

**Table 2.** Reproductive endocrine profiles of offspring born to mothers with and without poor ovarian response.

	POSEIDON-off groups				Non-POSEIDON-off groups	
	P1-off	P2-off	P3-off	P4-off	N1-off	N2-off
**Female offspring**	**n = 340 (453 visits)**	**n = 98 (134 visits)**	**n = 114 (158 visits)**	**n = 68 (92 visits)**	**n = 733 (968 visits)**	**n = 118 (151 visits)**
Age, years	2.9 ± 1	2.9 ± 1	3 ± 1.1	2.8 ± 0.9	2.9 ± 0.9	3 ± 1
FSH, IU/L	3.3 ± 1.8	3.4 ± 1.7	3.3 ± 2	3.3 ± 1.4	3.2 ± 1.6	2.9 ± 1.4
LH, IU/L^†^	0.1 ± 0	0.1 ± 0	0.1 ± 0	0.1 ± 0	0.1 ± 0	0.1 ± 0
E_2_, pg/ml	6.4 ± 3	6.2 ± 2.9	6.3 ± 5.1	5.7 ± 1.8	6.5 ± 4	6.2 ± 2.8
PRL, ng/L	12.5 ± 8.1	13.7 ± 8.9	12 ± 6	12.9 ± 8.2	13.2 ± 7.6	13.6 ± 9.3
T, ng/dl^†^	2.5 ± 0	2.5 ± 0	2.5 ± 0.2	2.5 ± 0	2.5 ± 0	2.5 ± 0
DHEA-S, ug/dl	7 ± 8.3	7.1 ± 8.2	8.3 ± 8.1	6.5 ± 7.6	7.9 ± 10.9	7.6 ± 7.8
AMH, ng/ml^¶^	2.9 ± 2	2.9 ± 1.8	2.4 ± 1.7	2.3 ± 1.7	3 ± 2.2	3.2 ± 2.2
**Male offspring**	**n = 335 (445 visits)**	**n = 102 (138 visits)**	**n = 152 (204 visits)**	**n = 76 (101 visits)**	**n = 846 (1187 visits)**	**n = 121 (158 visits)**
Age, years	2.9 ± 1	2.8 ± 0.9	2.9 ± 1	2.7 ± 0.8	2.9 ± 1	2.8 ± 0.9
FSH, IU/L	0.9 ± 0.6	0.9 ± 0.4	1 ± 0.5	0.9 ± 0.6	0.9 ± 0.4	0.8 ± 0.4
LH, IU/L	0.1 ± 0.1	0.1 ± 0.1	0.1 ± 0.1	0.1 ± 0.1	0.1 ± 0.1	0.1 ± 0.1
E_2_, pg/ml	5.4 ± 1.6	5.4 ± 1.3	5.4 ± 1.4	5.2 ± 1.2	5.4 ± 2.3	5.4 ± 1.4
PRL, ng/L	12.4 ± 7.3	11.2 ± 7.8	11.9 ± 5.7	11.6 ± 6.3	11.9 ± 6.4	12.5 ± 7.7
T, ng/dl^†^	2.5 ± 0	2.5 ± 0	2.5 ± 0	2.5 ± 0	2.5 ± 0	2.5 ± 0
DHEA-S, ug/dl	6.8 ± 9.3	7.5 ± 9.7	5.7 ± 7.3	6.4 ± 9.2	6.5 ± 9.7	6.4 ± 8
AMH, ng/ml^†^^,^^¶^	17.9 ± 0.6	18 ± 0.5	18 ± 0.1	18 ± 0.3	18 ± 0.5	18 ± 0.2

Data were presented as mean ± SD.

† The minimum detectable value for LH is 0.1 IU/L, and almost all female offspring have a detection value of <0.1 IU/L (96.6%). The minimum detectable value for T is 2.5 ng/dl, and almost all offspring have a detection value of <2.5 ng/dl (99.9%); the maximum detectable value for AMH is 18 ng/ml, and almost all male offspring have a detection value of >18 ng/dl (98.7%).

¶ For the AMH of female offspring, there are missing sample sizes of 54, 11, 27, 18, 138, and 27 in each group. For the AMH of male offspring, there are missing sample sizes of 52, 11, 28, 8, 130, and 14 in each group.

AMH, anti-Müllerian hormone; DHEA-S, dehydroepiandrosterone sulfate; E_2,_ estradiol; FSH, follicle-stimulating hormone; N1-off, offspring born to Non-POSEIDON Group 1 mothers; N2-off, offspring born to Non-POSEIDON Group 2 mothers; POSEIDON, Patient-Oriented Strategies Encompassing IndividualizeD Oocyte Number; P1-off, offspring born to POSEIDON Group 1 mothers; P2-off, offspring born to POSEIDON Group 2 mothers; P3-off, offspring born to POSEIDON Group 3 mothers; P4-off, offspring born to POSEIDON Group 4 mothers; PRL, prolactin; T, testosterone.

**Table 3.** Associations between maternal POR and the reproductive endocrine profile of female offspring.

		P1-off vs N1-off	P2-off vs N2-off	P3-off vs N1-off	P4-off vs N2-off
FSH	Crude MD (95% CI)	0.11 (−0.09, 0.31)	**0.43 (0.04, 0.82)** *	0.12 (−0.18, 0.42)	0.29 (−0.10, 0.69)
	Adjusted MD (95% CI)	0.12 (−0.07, 0.32)	0.37 (−0.01, 0.74)	0.18 (−0.12, 0.48)	0.22 (−0.15, 0.59)
E_2_	Crude MD (95% CI)	0.01 (−0.42, 0.43)	−0.03 (−0.70, 0.64)	−0.06 (−0.79, 0.67)	−0.49 (−1.14, 0.16)
	Adjusted MD (95% CI)	0.22 (−0.22, 0.66)	0.20 (−0.49, 0.89)	0.26 (−0.50, 1.01)	−0.43 (−1.11, 0.25)
PRL	Crude MD (95% CI)	−0.66 (−1.58, 0.27)	0.14 (−2.05, 2.33)	−1.30 (−2.63, 0.04)	−0.26 (−2.70, 2.17)
	Adjusted MD (95% CI)	−0.72 (−1.68, 0.24)	−0.03 (−2.24, 2.17)	−**1.59 (**−**2.97,** −0.21)*	−0.19 (−2.70, 2.32)
DHEA−S	Crude MD (95% CI)	−0.98 (−2.20, 0.25)	−0.43 (−2.52, 1.67)	0.35 (−1.61, 2.30)	−1.10 (−3.36, 1.16)
	Adjusted MD (95% CI)	−**1.38 (**−**2.58,** −0.17)*	−0.32 (−2.45, 1.81)	−0.41 (−2.36, 1.54)	−0.53 (−2.87, 1.81)
AMH	Crude MD (95% CI)	−0.10 (−0.38, 0.18)	−0.34 (−0.90, 0.23)	−**0.57 (**−**1.02,** −0.13)*	−**0.78 (**−**1.46,** −0.11)*
	Adjusted MD (95% CI)	−0.04 (−0.33, 0.25)	−0.16 (−0.75, 0.43)	−**0.64 (**−**1.10,** −0.18)**	−0.60 (−1.31, 0.12)

Crude mean differences were obtained from linear mixed regression analysis.

The adjusted model adjusted for paternal age, maternal age, offspring age, paternal smoking, ICSI use, and frozen embryo transfer use.

The reference group for the P1-off and P3-off groups was the N1-off group, whereas the reference group for the P2-off and P4-off groups was the N2-off group.

LH and testosterone (T) were not in detectable range among nearly all female offspring, so they could not be analyzed statistically.

Bold font is used to highlight statistically significant difference between groups, with a *P*-value of less than 0.05.

* *P *<* *0.05,

** *P *<* *0.01.

AMH, anti-Müllerian hormone; DHEA-S, dehydroepiandrosterone sulfate; E_2,_ estradiol; MD, mean difference; N1-off, offspring born to Non-POSEIDON Group 1 mothers; N2-off, offspring born to Non-POSEIDON Group 2 mothers; POSEIDON, Patient-Oriented Strategies Encompassing IndividualizeD Oocyte Number; P1-off, offspring born to POSEIDON Group 1 mothers; P2-off, offspring born to POSEIDON Group 2 mothers; P3-off, offspring born to POSEIDON Group 3 mothers; P4-off, offspring born to POSEIDON Group 4 mothers; POR, poor ovarian response; PRL, prolactin.

Daughters born to mothers in POSEIDON Group 1 displayed lower DHEA-S levels in the adjusted model (MD = −0.98, 95% CI = −2.20, 0.25, *P* = 0.117; AMD = −1.38, 95% CI = −2.58, −0.17, *P* = 0.025) (Tables 2 and 3 and Supplementary Table S2).

For male offspring, reproductive endocrine profiles are presented in Tables 2 and 4 and Supplementary Table S3. No differences were found in FSH, LH, E_2_, PRL, and DHEA-S levels between male offspring born to mothers with unexpected POR and those born to mothers without POR.

**Table 4.** Associations between maternal POR and the reproductive endocrine profile of male offspring.

		P1-off vs N1-off	P2-off vs N2-off	P3-off vs N1-off	P4-off vs N2-off
FSH	Crude MD (95% CI)	0.03 (−0.03, 0.08)	0.05 (−0.06, 0.16)	0.05 (−0.02, 0.12)	0.09 (−0.05, 0.23)
	Adjusted MD (95% CI)	0.03 (−0.03, 0.09)	0.06 (−0.05, 0.17)	0.05 (−0.02, 0.13)	0.09 (−0.06, 0.24)
LH	Crude MD (95% CI)	0.004 (−0.005, 0.01)	0.01 (−0.01, 0.03)	0.01 (−0.003, 0.02)	0.01 (−0.02, 0.04)
	Adjusted MD (95% CI)	0.01 (−0.004, 0.02)	0.01 (−0.02, 0.03)	0.01 (−0.003, 0.02)	0.01 (−0.02, 0.05)
E_2_	Crude MD (95% CI)	−0.02 (−0.29, 0.25)	0.01 (−0.31, 0.34)	−0.03 (−0.41, 0.34)	−0.12 (−0.46, 0.21)
	Adjusted MD (95% CI)	−0.03 (−0.32, 0.25)	−0.04 (−0.38, 0.30)	−0.02 (−0.40, 0.37)	−0.22 (−0.60, 0.17)
PRL	Crude MD (95% CI)	0.57 (−0.21, 1.34)	−1.03 (−2.93, 0.88)	−0.06 (−1.05, 0.93)	−0.97 (−2.87, 0.94)
	Adjusted MD (95% CI)	0.67 (−0.14, 1.48)	−0.93 (−2.96, 1.11)	−0.11 (−1.12, 0.90)	−0.48 (−2.61, 1.65)
DHEA-S	Crude MD (95% CI)	0.47 (−0.66, 1.60)	1.63 (−0.63, 3.89)	−0.60 (−2.11, 0.91)	0.03 (−2.26, 2.32)
	Adjusted MD (95% CI)	0.51 (−0.62, 1.63)	1.56 (−0.77, 3.88)	−0.86 (−2.33, 0.60)	0.10 (−2.29, 2.48)

Crude mean differences were obtained from linear mixed regression analysis.

The adjusted model adjusted for paternal age, maternal age, offspring age, paternal smoking, ICSI use, and frozen embryo transfer use.

The reference group for the P1-off and P3-off groups was the N1-off group, whereas the reference group for the P2-off and P4-off groups was the N2-off group.

Testosterone (T) and anti-Müllerian hormone (AMH) were not in detectable range among nearly all male offspring, so they could not be analyzed statistically.

DHEA-S, dehydroepiandrosterone sulfate; E_2,_ estradiol; MD, mean difference; N1-off, offspring born to Non-POSEIDON Group 1 mothers; N2-off, offspring born to Non-POSEIDON Group 2 mothers; POSEIDON, Patient-Oriented Strategies Encompassing IndividualizeD Oocyte Number; P1-off, offspring born to POSEIDON Group 1 mothers; P2-off, offspring born to POSEIDON Group 2 mothers; P3-off, offspring born to POSEIDON Group 3 mothers; P4-off, offspring born to POSEIDON Group 4 mothers; POR, poor ovarian response; PRL, prolactin.

### The effects of maternal expected POR on reproductive endocrine characteristics of offspring

Female offspring born to mothers in POSEIDON Group 3 had significantly lower levels of AMH compared to their counterparts in both unadjusted and adjusted models (MD = −0.57, 95% CI = −1.02, −0.13, *P* = 0.012; AMD = −0.64, 95% CI = −1.10, −0.18, *P* = 0.007), with statistical power reaching 95% (data not shown). Female offspring born to mothers in POSEIDON Group 4 also showed lower levels of AMH in the unadjusted model (MD = −0.78, 95% CI = −1.46, −0.11, *P* = 0.023). Although the trend remained the same, the lack of statistical significance in the adjusted model may be due to limitations in sample size (AMD = −0.60, 95% CI = −1.31, −0.12, *P* = 0.100) (Tables 2 and 3 and Supplementary Table S2).

Compared to the control group, daughters born to mothers in POSEIDON Group 3 had lower PRL levels in the adjusted model (MD = −1.30, 95% CI = −2.63, 0.04, *P* = 0.057; AMD = −1.59, 95% CI = −2.97, −0.21, *P* = 0.024) (Tables 2 and 3 and Supplementary Table S2).

For male offspring, no differences in reproductive endocrine profiles were observed between those born to mothers with expected POR and those born to controls (Tables 2 and 4 and Supplementary Table S3).

## Discussion

In this study, we found a trend for modest decrease in AMH levels in daughters born to expected POR mothers aged 2–6 years (POSEIDON Group 3 and Group 4). However, only female offspring born to young mothers (<35 years) with expected POR (POSEIDON Group 3) showed a statistically significant difference, while those born to older mothers (≥35 years) with expected POR (POSEIDON Group 4) did not exhibit a significant difference. These results potentially indicate an intergenerational effect on ovarian reserve. Nevertheless, no significant association was observed between maternal POR and the reproductive endocrine profiles of male offspring at an early age. Additionally, we found that mothers with POR had lower T levels during the first trimester of pregnancy.

Two previous cross-sectional studies reported a significant adverse effect of earlier age at maternal menopause on both serum AMH and AFC in daughters during reproductive age (Rosen *et al.*, 2010; Bentzen *et al.*, 2013), which are consistent with our findings. The underlying mechanisms remain unclear, but genetic susceptibility and epigenetic modification were believed to be the main reason (Cavalli and Heard, 2019). Previous studies have identified some mutations and polymorphic variant genes involved in folliculogenesis and follicular atresia, which play a role in pathologic expected POR (Nesbit *et al.*, 2020; Olsen *et al.*, 2021). These mutations may potentially be transmitted to the next generation. Furthermore, other studies suggested that epigenetic modifications, including changes in DNA methylation, the involvement of non-coding RNAs, and RNA m6A modifications, may contribute to pathologic expected POR by regulating oocyte development, maturation, growth, and senescence (Olsen *et al.*, 2021; Chen *et al.*, 2025). Epigenetic changes in oocytes have been shown to affect offspring health across generations through intergenerational or transgenerational inheritance (Chen *et al.*, 2022a). These findings suggest that both genetic and epigenetic factors could have significant implications for reproductive health and the inheritance of fertility traits across generations.

Moreover, decreased T levels in mothers with expected POR during pregnancy may also contribute to the decrease in AMH levels in female offspring. Previous studies have demonstrated that a hypoandrogenic intrauterine environment may cause ovarian damage in the offspring (Yu *et al.*, 2021). In contrast, prenatal testosterone excess has been shown to increase follicular recruitment in female sheep offspring (Steckler *et al.*, 2005; Smith *et al.*, 2009). Additionally, other environmental factors may provide another explanation. Growing evidence has suggested that environmental exposure may contribute to poor ovarian reserve, including toxins, stress, and certain medical treatments (Richardson *et al.*, 2014; Wang *et al.*, 2021; Xi *et al.*, 2022). These exposures during early life were found to affect follicular development in offspring in some animal studies (Jurisicova *et al.*, 2007; Liu *et al.*, 2021; Chen *et al.*, 2022b; Yan *et al.*, 2022). Further research is needed to elucidate the mechanisms underlying the intergenerational transmission of reduced ovarian reserve.

In our study, we observed that while AMH levels in daughters born to older mothers with expected POR (POSEIDON Group 4) showed a decreasing trend; this difference was not significant after adjusting for confounders. In contrast to young women with expected POR, ovarian reserve reduction in older women is more likely a consequence of physiological aging (Lu *et al.*, 2023). Previous studies have reported reduced oocyte developmental potential, mitochondrial dysfunction, and impaired oxidative phosphorylation in cumulus cells in older women with expected POR (Cecchino *et al.*, 2021; Lu *et al.*, 2022; Almeida-Reis *et al.*, 2024). In our own observations, these age-related changes in mothers did not seem to affect the ovarian reserve of their daughters.

However, it is important to note that the smaller number of offspring in the older group limits our statistical power, which may affect our findings. Although the prevalence of POR in older women is similar to that in younger groups (Haahr *et al.*, 2019; Esteves *et al.*, 2021a), studies have shown that advanced maternal age correlates with lower CLBR in ART (Luke *et al.*, 2012; Gaskins *et al.*, 2023; Xia *et al.*, 2024). CLBR in younger women with POR is more than twice as high as in older women (Esteves *et al.*, 2021b; Li *et al.*, 2021). Thus, the smaller sample size in the older group may have influenced our findings, particularly with regard to AMH changes in female offspring born to older mothers with expected POR.

AMH, the most important marker of ovarian reserve, is secreted by the granulosa cells of preantral follicles as early as 36 weeks’ gestation (Rajpert-De Meyts *et al.*, 1999; Cui *et al.*, 2016). In our study, female offspring born to young mothers with expected POR exhibited a 0.64 ng/ml decrease in AMH levels compared to the control group at ages 2–6. Previous longitudinal studies reported that AMH levels in the prepubertal period were positively associated with ovarian reserve later in life (Brougham *et al.*, 2012; Hagen *et al.*, 2012). The reduced AMH levels observed in our study suggest that the female offspring may possess a relatively small follicular pool and potentially lower quality of oocytes, raising concerns about fertility and ovarian aging in their reproductive years.

In clinical practice, elevated serum AMH levels are linked to a higher number of oocytes retrieved and more cryopreserved embryos, which contributes to a higher pregnancy rate (Peigné *et al.*, 2023). AMH testing is also useful for predicting POR, cycle cancellations, and the risk of ovarian hyperstimulation syndrome (La Marca *et al.*, 2010). A prospective study confirmed that an AMH threshold of 4 pmol/L (0.56 ng/ml) can predict a low yield of mature follicles and oocytes (La Marca *et al.*, 2010). However, there is currently no established clinical threshold for AMH levels in female offspring during childhood. As AMH levels naturally decline with age after 25 years old (Lie Fong *et al.*, 2012; Cui *et al.*, 2016), long-term follow-up and fertility monitoring are important, particularly for this population.

Consistent with other studies, our research also found that women with POR often exhibit disorders in the hypothalamus–pituitary–ovarian (HPO) axis, including elevated FSH, decreased LH, and decreased T (Zhen *et al.*, 2008; Xiao *et al.*, 2016; Zhuang *et al.*, 2019). Additionally, our study observed significantly lower PRL levels in young women with expected POR (POSEIDON Group 3). The HPO axis remains quiescent between minipuberty (1–6 months) and puberty (Renault *et al.*, 2020), and previous epidemiological studies have shown that sex hormone levels in females remain low and stable during this period (Soldin *et al.*, 2005; Konforte *et al.*, 2013; Tahmasebi *et al.*, 2017; Bohn *et al.*, 2021). In our study, we could not detect significant symbolic changes in gonadotropin and steroid hormones in the female offspring with maternal POR. However, an increasing trend in FSH levels was noted across female offspring born to all types of POR mothers based on the POSEIDON classification, although these differences did not reach statistical significance in our study. Additionally, levels of DHEA-S, a precursor to T, were found to be decreased in daughters born to young mothers with unexpected POR (POSEIDON Group 1). PRL levels were also found to be decreased in daughters born to young mothers with expected POR (POSEIDON Group 3).

Young women with unexpected POR typically have the best prognosis compared to other POSEIDON groups (Alviggi *et al.*, 2016). Management requires a tailored approach considering the patient’s young age and adequate ovarian reserve, potentially involving higher starting doses of recombinant FSH, recombinant LH supplementation, or even double ovarian stimulation to enhance oocyte retrieval and improve reproductive outcomes (Conforti *et al.*, 2019; Polyzos and Drakopoulos, 2019). The high doses of gonadotropins administered to the oocytes that develop into embryos during both fresh and frozen cycles, along with the uterine hormonal environment, which is present only during fresh cycles, may potentially explain the changes observed in DHEA-S levels in female offspring. These findings indicate that there may be pituitary dysfunction in this specific population. For female offspring born to young mothers with unexpected POR, genetic factors, including mutations or polymorphisms, epigenetic modifications, and environmental factors, such as a hypoandrogenic intrauterine environment and other factors, may contribute to the concordant changes in PRL levels between mothers and their daughters. However, due to relatively limited statistical power, the statistical powers of DHEA-S and PRL changes were less than 80% (data not shown). Larger sample sizes and longer follow-up studies are required to confirm these findings.

Though previous studies reported that basal serum T levels decreased in POR women compared to normal responders, there is no evidence of its continuation or worsening progression after conception (Woldringh *et al.*, 2006; Qin *et al.*, 2011). Our study indicated that even at the end of the first trimester, characteristic deregulation of serum T persisted in mothers with POR. It was suggested that deficiency of prenatal androgen exposure may affect the masculinization of the genitalia in male offspring, including increased risk of hypospadias and cryptorchidism, as well as a decreased anogenital distance (Menon and Khatwa, 2000; Domínguez-Salazar *et al.*, 2002; Welsh *et al.*, 2008; Sunman *et al.*, 2019). Additionally, reduced intrauterine exposure to T can lead to behavioral and metabolic changes in offspring, including reduced aggression, motor activity, and insulin secretion (Goto *et al.*, 2005; Harada *et al.*, 2019; Drea *et al.*, 2021). Encouragingly, our study found no significant effects on the reproductive endocrine profiles of male offspring with maternal POR up to 6 years of age. However, the reproductive function (specifically serum endocrine and seminal fluid analysis), behavioral patterns, and metabolism of these boys still should be further evaluated and monitored in later life.

To the best of our knowledge, the health of offspring born to mothers with POR has historically been a concern because of suspected genetic susceptibility, deteriorating oocyte quantity, and intrauterine exposure. However, this concern has yet to be confirmed. We used a large cohort study to provide valuable evidence for our question of interest. Furthermore, mothers with POR were stratified into groups based on the POSEIDON criteria, which can be beneficial for understanding the effects of maternal POR under different pathophysiological conditions. The observers were well-trained pediatricians, nurses, and laboratory physicians, rather than clinicians from infertility clinics, and they were blinded to the exposure status of the participants, which helps to avoid potential selection bias in the study. In addition, the data were highly homogeneous due to standardized operating procedure for all participants and tests.

However, there are still some limitations. Firstly, at the time of our study, the offspring were between the ages of 2 and 6 years. As they are too young to reveal more comprehensive reproductive phenotypic changes, longer follow-up studies are needed. Secondly, the confounding factors could not be addressed completely in our retrospective study. In the past few decades, various adjuvant therapies were proposed to improve success rate in POR patients (Zhang *et al.*, 2020), but we cannot exclude the potential impact of such therapies on offspring health. Nonetheless, our results still have valuable clinical relevance. Thirdly, maternal smoking during pregnancy was not included in this study, which could be a potential confounder. Previous research has shown that maternal tobacco exposure adversely affects the fertility of both female and male offspring (Ernst *et al.*, 2012; Sobinoff *et al.*, 2014). However, only 3.4% of Chinese women were reported as smokers in the 2010 national survey (Liu *et al.*, 2017), with approximately 82.9% quitting upon pregnancy (Xu *et al.*, 2017). Consequently, maternal smoking during pregnancy is extremely rare in China, and this potential bias is unlikely to significantly influence our findings. Additionally, not all offspring who met our inclusion criteria in the ‘ARTKID’ cohort provided blood samples. Possible reasons include failure to meet follow-up deadlines, noncompliance with invasive blood testing, and external factors such as the COVID-19 pandemic during 2020–2021, as well as geographical distance from our center. We compared the baseline characteristics between the included and excluded offspring and found that the differences were relatively small. Therefore, we conclude that selection bias is unlikely to have a significant impact on our results.

## Conclusions

In conclusion, the potential impact of maternal POR on offspring appears to be influenced by the ovarian reserve in mothers. Young mothers (<35 years) with expected POR were more likely to have daughters with slightly lower serum AMH levels, whereas other POR mothers seem to pose less risk to reproductive endocrine health of their offspring. Longer-term observations on a larger scale are needed to confirm these findings and to further elucidate the underlying mechanism.

## Supplementary Material

hoaf019_Supplementary_Data

## Data Availability

The data set cannot be shared directly due to current data protection legislation. Access to the data can be requested from the Birth Cohort Data Management Committee at the State Key Laboratory of Reproductive Medicine and Offspring Health, Center for Reproductive Medicine, Institute of Women, Children and Reproductive Health, Shandong University (ARTKID_sdu@163.com), following approval from the ethics committees at the Reproductive Medical Center of Shandong University (wanghuidan@sduivf.com).
