# Supplementary material for: Effects of maternal poor ovarian response on the reproductive endocrine profiles of the next generation: a prospective cohort study in China
Source: Hum Reprod Open. 2025 Mar 28;2025(2):hoaf019. doi: 10.1093/hropen/hoaf019 (PMC12080958; doi:10.1093/hropen/hoaf019)
Supplement: hoaf019_Supplementary_Data [file hoaf019_supplementary_data.zip › Supplemental-Tables-post adjudication clean.docx]

**Supplementary Table S1** Characteristics of included and excluded study children.

|  | **Excluded-off**  **n=6380** | **Included-off**  **n=3103** | **MD or OR**  **(95% CI)** | **P-value** |
| --- | --- | --- | --- | --- |
| Paternal age, years | 32.2±5 | 31.7±5 | **-0.53 (-0.74, -0.31)** | **<0.001** |
| Maternal age, years | 31.4±4.3 | 31±4.4 | **-0.44 (-0.63, -0.26)** | **<0.001** |
| Paternal BMI, kg/m2 | 26±4.1 | 26±4 | -0.02 (-0.19, 0.15) | 0.077 |
| Maternal BMI, kg/m2 | 23.2±3.5 | 23.3±3.6 | 0.14 (-0.02, 0.29) | 0.828 |
| Paternal education level (college or higher), n (%) | 2599 (40.7) | 1246 (40.2) | 0.98 (0.89, 1.07) | 0.588 |
| Maternal education level (college or higher), n (%) | 2397 (37.6) | 1127 (36.3) | 0.95 (0.87, 1.04) | 0.237 |
| Paternal smoking, n (%) | 2180 (34.2) | 996 (32.1) | **0.91 (0.83, 0.998)** | **0.045** |
| Maternal AFC | 12.7±5.8 | 13±5.9 | **0.36 (0.11, 0.61)** | **0.005** |
| Maternal basal endocrine | |  |  |  |
| AMH, ng/mL | 4.4±3.5 | 4.3±3.4 | -0.13 (-0.28, 0.02) | 0.095 |
| FSH, IU/L | 6.8±2.5 | 6.8±2.1 | -0.07 (-0.17, 0.04) | 0.203 |
| LH, IU/L | 5.2±2.9 | 5.1±2.8 | -0.05 (-0.17, 0.07) | 0.443 |
| E2, pg/mL | 36.5±17.9 | 36.1±19.3 | -0.47 (-1.26, 0.33) | 0.252 |
| T, ng/dL | 24.4±14 | 24.1±12.4 | -0.22 (-0.8, 0.36) | 0.454 |
| PRL, ng/L | 17.3±13.6 | 17.4±12.2 | 0.11 (-0.45, 0.68) | 0.716 |
| DHEA-S, ug/dL | 243.6±91.6 | 243.7±92 | 0.09 (-4.11, 4.29) | 0.966 |
| Maternal total gonadotropin dose, kIU | 1.8±0.9 | 1.9±0.9 | 0.03 (-0.003, 0.07) | 0.072 |
| Maternal E2 levels on hCG trigger day, ng/mL | 3.5±0.2 | 3.7±0.2 | **0.13 (0.05, 0.22)** | **0.003** |
| No. of oocytes retrieved | 11.5±5.6 | 11.9±5.8 | **0.42 (0.18, 0.67)** | **0.001** |

**Note:** Data were presented as median (interquartile range) or n (%).

Bold font is used to highlight statistically significant difference between the two groups, with a p-value of less than 0.05.

**Abbreviations:** AFC, antral follicle count; AMH, anti-Müllerian hormone; DHEA-S, dehydroepiandrosterone sulfate; E_2_, estradiol; MD, mean difference; OR, odds ratio; PRL, prolactin; T, testosterone.

**Supplementary Table S2.** Differential p-values for Reproductive Endocrine Variables in Female Offspring Born to Mothers with and without POR.

|  |  | **P1-off vs. N1-off** | **P2-off vs. N2-off** | **P3-off vs. N1-off** | **P4-off vs. N2-off** |
| --- | --- | --- | --- | --- | --- |
| FSH | Crude P | 0.272 | **0.031** | 0.424 | 0.144 |
|  | Adjusted P | 0.225 | 0.054 | 0.237 | 0.249 |
| E_2_ | Crude P | 0.975 | 0.931 | 0.869 | 0.141 |
|  | Adjusted P | 0.326 | 0.571 | 0.503 | 0.211 |
| PRL | Crude P | 0.165 | 0.900 | 0.057 | 0.832 |
|  | Adjusted P | 0.139 | 0.976 | **0.024** | 0.880 |
| DHEA-S | Crude P | 0.117 | 0.690 | 0.729 | 0.338 |
|  | Adjusted P | **0.025** | 0.769 | 0.678 | 0.656 |
| AMH | Crude P | 0.496 | 0.242 | **0.012** | **0.023** |
|  | Adjusted P | 0.790 | 0.600 | **0.007** | 0.100 |

**Note:** Crude mean differences were obtained from linear mixed regression analysis.

The adjusted model adjusted for paternal age, maternal age, offspring age, paternal smoking, ICSI use, and frozen embryo transfer use.

Luteinizing hormone (LH) and testosterone (T) were not in detectable range among nearly all female offspring, so they could not be analyzed statistically.

Bold font is used to highlight statistically significant difference between groups, with a p-value of less than 0.05.

**Abbreviations:** AMH, anti-Müllerian hormone; DHEA-S, dehydroepiandrosterone sulfate; E_2,_ estradiol; MD, mean difference; N1-off, offspring born to Non-POSEIDON Group 1 mothers; N2-off, offspring born to Non-POSEIDON Group 2 mothers; POSEIDON, Patient-Oriented Strategies Encompassing IndividualizeD Oocyte Number; P1-off, offspring born to POSEIDON Group 1 mothers; P2-off, offspring born to POSEIDON Group 2 mothers; P3-off, offspring born to POSEIDON Group 3 mothers; P4-off, offspring born to POSEIDON Group 4 mothers; POR, poor ovarian response; PRL, prolactin; T, testosterone.

**Supplementary Table S3.** Differential p-values for Reproductive Endocrine Variables in Male Offspring Born to Mothers with and without POR.

|  |  | **P1-off vs. N1-off** | **P2-off vs. N2-off** | **P3-off vs. N1-off** | **P4-off vs. N2-off** |
| --- | --- | --- | --- | --- | --- |
| FSH | Crude P | 0.382 | 0.343 | 0.131 | 0.201 |
|  | Adjusted P | 0.374 | 0.306 | 0.133 | 0.227 |
| LH | Crude P | 0.412 | 0.562 | 0.145 | 0.421 |
|  | Adjusted P | 0.270 | 0.642 | 0.126 | 0.422 |
| E_2_ | Crude P | 0.867 | 0.938 | 0.874 | 0.471 |
|  | Adjusted P | 0.822 | 0.813 | 0.928 | 0.266 |
| PRL | Crude P | 0.150 | 0.291 | 0.905 | 0.319 |
|  | Adjusted P | 0.105 | 0.370 | 0.831 | 0.659 |
| DHEA-S | Crude P | 0.426 | 0.156 | 0.434 | 0.980 |
|  | Adjusted P | 0.381 | 0.188 | 0.248 | 0.937 |

**Note:** Crude mean differences were obtained from linear mixed regression analysis.

The adjusted model adjusted for paternal age, maternal age, offspring age, paternal smoking, ICSI use, and frozen embryo transfer use.

Testosterone (T) and anti-Müllerian hormone (AMH) were not in detectable range among nearly all male offspring, so they could not be analyzed statistically.

**Abbreviations:** AMH, anti-Müllerian hormone; DHEA-S, dehydroepiandrosterone sulfate; E_2,_ estradiol; MD, mean difference; N1-off, offspring born to Non-POSEIDON Group 1 mothers; N2-off, offspring born to Non-POSEIDON Group 2 mothers; POSEIDON, Patient-Oriented Strategies Encompassing IndividualizeD Oocyte Number; P1-off, offspring born to POSEIDON Group 1 mothers; P2-off, offspring born to POSEIDON Group 2 mothers; P3-off, offspring born to POSEIDON Group 3 mothers; P4-off, offspring born to POSEIDON Group 4 mothers; POR, poor ovarian response; PRL, prolactin; T, testosterone.
